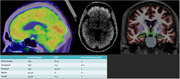# Progressive Dysexecutive Syndrome (PDS) due to Alzheimer’s Disease: Genetic and Neuroinflammatory Correlates in a Rapidly Progressive Case

**DOI:** 10.1002/alz.092098

**Published:** 2025-01-03

**Authors:** Aaron Ritter, Kaley Brouwers, Jefferson W Kinney

**Affiliations:** ^1^ Hoag Medical Center, Newport Beach, CA USA; ^2^ Cleveland Clinic Neurosciences Institute, Las Vegas, NV USA; ^3^ University of Nevada Las Vegas, Las Vegas, NV USA

## Abstract

**Background:**

Many patients present to our clinic with primarily executive rather than amnestic impairments. Recently, Townley and colleagues proposed criteria for a progressive dysexecutive syndrome (PDS). To date, PDS has been reported to be more common in younger individuals (55‐65 years old) and is associated with Alzheimer’s biomarkers (AD). Less is known about the genetic and neuroinflammatory profile of patients with PDS.

**Method:**

We report the clinical, biomarker, genetic, and autopsy data of a participant from the Center for Translational and Neuroscience (CNTN) meeting criteria for PDS.

**Result:**

A 57‐year‐old left‐handed male presented with primary executive difficulties (bill paying, financial difficulties). Apathy was the only behavioral symptom reported. His initial neuropsychological testing showed impairments across a broad range of executive measures which worsened significantly over three years. Biomarker testing was consistent with Alzheimer’s disease (A+T+N+) with a significant elevation in plasma p‐tau 181 and neuroimaging showing peak involvement of the parietal lobes. Several plasma markers of neuroinflammation were elevated (GFAP, NFL) while others were depressed (IL‐6, IL‐10, TNF‐alpha). He was negative for autosomal dominant mutations (PS1, PS2, APP) but carried two apolipoprotein E4 genes (APOE 4/4) with an elevated polygenic risk score. Exome sequencing revealed polymorphisms within several known risk genes: CR1, PICALM, ABCA7. After experiencing a rapid cognitive decline, the participant died only five years after the onset of symptoms. Due to sparse neurofibillary tanges in the mesial temporal cortex he was characterized as having “hippocampal sparring” Alzheimer’s disease.

**Conclusion:**

Progressive dysexecutive syndrome is a newly described variant of AD that affects younger individuals, causes impairments in executive functioning, and primarily impacts the fronto‐parietal networks. Findings from our case report suggest a possible neuroinflammatory susceptibility and multi‐factorial genetic basis.